# Cellular Hypertrophy and Increased Susceptibility to Spontaneous Calcium-Release of Rat Left Atrial Myocytes Due to Elevated Afterload

**DOI:** 10.1371/journal.pone.0144309

**Published:** 2015-12-29

**Authors:** Haifei Zhang, Mark B. Cannell, Shang Jin Kim, Judy J. Watson, Ruth Norman, Sarah C. Calaghan, Clive H. Orchard, Andrew F. James

**Affiliations:** 1 Cardiovascular Research Laboratories, School of Physiology, Pharmacology & Neuroscience, University of Bristol, Bristol, BS8 1TD, United Kingdom; 2 School of Biomedical Sciences, Garstang, University of Leeds, Leeds, LS2 9JT, United Kingdom; University of Canberra, AUSTRALIA

## Abstract

Atrial remodeling due to elevated arterial pressure predisposes the heart to atrial fibrillation (AF). Although abnormal sarcoplasmic reticulum (SR) function has been associated with AF, there is little information on the effects of elevated afterload on atrial Ca^2+^-handling. We investigated the effects of ascending aortic banding (AoB) on Ca^2+^-handling in rat isolated atrial myocytes in comparison to age-matched sham-operated animals (Sham). Myocytes were either labelled for ryanodine receptor (RyR) or loaded with fluo-3-AM and imaged by confocal microscopy. AoB myocytes were hypertrophied in comparison to Sham controls (P<0.0001). RyR labeling was localized to the z-lines and to the cell edge. There were no differences between AoB and Sham in the intensity or pattern of RyR-staining. In both AoB and Sham, electrical stimulation evoked robust SR Ca^2+^-release at the cell edge whereas Ca^2+^ transients at the cell center were much smaller. Western blotting showed a decreased L-type Ca channel expression but no significant changes in RyR or RyR phosphorylation or in expression of Na^+^/Ca^2+^ exchanger, SR Ca^2+^ ATPase or phospholamban. Mathematical modeling indicated that [Ca^2+^]_i_ transients at the cell center were accounted for by simple centripetal diffusion of Ca^2+^ released at the cell edge. In contrast, caffeine (10 mM) induced Ca^2+^ release was uniform across the cell. The caffeine-induced transient was smaller in AoB than in Sham, suggesting a reduced SR Ca^2+^-load in hypertrophied cells. There were no significant differences between AoB and Sham cells in the rate of Ca^2+^ extrusion during recovery of electrically-stimulated or caffeine-induced transients. The incidence and frequency of spontaneous Ca^2+^-transients following rapid-pacing (4 Hz) was greater in AoB than in Sham myocytes. In conclusion, elevated afterload causes cellular hypertrophy and remodeling of atrial SR Ca^2+^-release.

## Introduction

Atrial fibrillation (AF) is the most common sustained arrhythmia, represents a major risk factor for stroke and is associated with high morbidity and mortality[1]. The most prevalent risk factor for the development of AF is hypertension[1, 2]. Animal models have shown that hypertension and elevated afterload cause structural and cellular remodeling of the atria that establishes a substrate for re-entrant arrhythmias[3–7]. However, the trigger mechanism(s) initiating AF are less clear[1] although abnormalities in Ca^2+^ handling are likely to play an important role[8–11]. In addition, tachyarrhythmia-induced electrical remodeling in the form of reduced atrial effective refractory period (AERP)[12], linked to reduced L-type Ca^2+^ channel (LTCC) current (*I*_*CaL*_) density and increased inward-rectifier K^+^ currents[13–15] contribute to the stabilization of the arrhythmia.

Atrial myocytes from AF patients show abnormal sarcoplasmic reticulum (SR) Ca^2+^ handling and an increased rate of spontaneous diastolic Ca^2+^ release compared with myocytes from patients in sinus rhythm[9–11]. Spontaneous diastolic release of SR Ca^2+^ can cause delayed afterdepolarizations (DADs), which constitute a major mechanism underlying focal triggered activity[8]. Thus, the AF-associated remodeling of Ca^2+^ handling is consistent with an increased susceptibility to triggered activity in atria from AF patients[8, 12]. Atrial myocytes from AF patients show complex and variable patterns of intracellular Ca^2+^ transport[8], but the extent to which this is altered in afterload-induced remodeling prior to the development of AF is unknown. Propagating Ca^2+^-induced Ca^2+^ release, which is known to be arrhythmogenic in ventricular myocytes[16], depends on the microscopic cellular architecture[17, 18] and might be altered during hypertension-induced cellular remodeling. We have examined this possibility by analyzing cell geometry, ryanodine receptor labeling and the spread of Ca^2+^ signals across cells from sham-operated and elevated afterload (aortic-banded) animals[4].

## Materials and Methods

All procedures were conducted in accordance with the Animals (Scientific Procedures) Act 1986 of the United Kingdom and were approved by the Research Ethics Committee of the University of Bristol. Surgical procedures were conducted under general anaesthesia (80 mg/kg ketamine and 8 mg/kg xylazine).

### Animal model with elevated afterload

A gradual increase in left ventricular afterload was achieved by partial stenosis of the ascending aorta in weanling rats, as described previously[4]. Briefly, 32 male Wistar rats (B&K Universal Ltd, UK), body weight 100–120 g (3–4 weeks of age), were subject to general anesthesia (80 mg/kg ketamine and 8 mg/kg xylazine), intubated and ventilated and a right thoracotomy performed. In 16 of these (AoB), a silk ligature (3–0) was tied around the ascending aorta to the outer diameter of a blunt 20-gauge needle (0.91 mm). The remaining 16 time-matched sham-operated controls (Sham) were subject to the same procedure except that a suture was not tied around the aorta. Experiments were conducted 20±1 weeks post-surgery.

### Cell isolation and storage

Left atrial myocytes were isolated from AoB and Sham hearts by Langendorff perfusion of the heart with a collagenase-containing solution and stored in Kraftbrühe (KB) solution at ~4°C until use, as described previously[3]. Cells were stored in Kraftbrühe (KB) solution containing (in mM), 70 L-glutamic acid, 30 KCl, 10 HEPES, 1 EGTA, 5 MgCl_2_, 5 Na-pyruvate, 20 taurine, 10 D-glucose, 5 succinic acid, 5 creatine, 2 Na_2_ATP, and 5 *β*-hydroxybutyric acid (pH 7.2) in a refrigerator (~4°C)[19].

### Confocal microscopy

Images were obtained from live and fixed cells using a laser scanning confocal microscope (LSM 510, Zeiss, Germany). The confocal aperture was set so that the confocal plane was ≤1 μm with a ×63 oil-immersion objective lens (Plan-Neofluar, numerical aperture 1.2, Zeiss, Germany).

### Cell labeling

To visualize the cell membrane, left atrial myocytes were stained with 5 μM di-8-ANEPPS (Invitrogen, UK) at room temperature (RT) for 5 minutes. After removal of unloaded dye by centrifugation (400 rpm, 40 s) and re-suspension in dye-free Tyrode’s solution, di-8-ANEPPS-loaded cells were transferred to a bath mounted on the LSM 510 (Zeiss, Germany). To examine the expression and localization of ryanodine receptors (RyR), myocytes were allowed to settle on poly-l-lysine coverslips for 1 h. Cells were fixed in 4% paraformaldehyde (PFA) for 10 min and subsequently permeabilized using 0.1% Triton-X for 10 min at RT. Fixed and permeabilized cells were incubated with 5 μg/ml of RyR antibody (Pierce Antibodies, MA3-916) for 1 h followed by incubation with 2 μg/ml Alexa Fluor 488-conjugated anti-mouse secondary antibody (Molecular Probes, A21121) for 1 h. Images were analyzed using ImageJ (NIH) and using routines written in IDL (Boulder, CO). The periodicity of the staining was investigated by obtaining the fluorescence profile along the longitudinal axis of the cell. The power spectrum of the fluorescence profile was calculated using the fast Fourier transform (FFT) and a Gaussian function was fitted to the first harmonic (IgorPro 3.16B, Wavemetrics Inc., OR). The spatial frequency corresponding to the peak of the power spectrum gave a measure of the periodic interval and the amplitude of the peak normalized as a percentage of the frequency-independent component of the spectrum provided a measure of the intensity of staining associated with the striations[20].

### Western blotting

10 μg samples of tissue homogenates were run on 6% reducing SDS-PAGE gels and transferred onto Immobilon-P membrane. Blots were probed with anti-ryanodine receptor 2 (anti-RyR2; MA3916, Thermo Scientific, UK), S2808-specific anti-phospho-RyR2 (2808 AP, Badrilla, UK), S2814-specific anti-phospho-RyR2 (A010-31AP, Badrilla, UK), anti-α_1c_ LTCC subunit (ACC-003; Alomone, Israel), anti-SR Ca^2+^ ATPase (anti-SERCA2; MA3-919, Thermo Scientific, UK), anti-phospholamban (anti-PLB; A010-14, Badrilla, UK), S16-specific anti-phosphorylated PLB (A010-12AP, Badrilla, UK), anti-Na^+^/Ca^2+^ exchanger (anti-NCX1; R3F1, Swannt, Switzerland) or anti-GAPDH (G9545; Sigma, UK) and protein bands visualized using relevant peroxidase-conjugated secondary antibodies, chemiluminescence and autoradiography. The S2808- and S2814-specific anti-phospho-RyR2 antibodies were used to investigate changes in RyR2 phosphorylation because phosphorylation at these sites for protein kinase A (PKA) and Ca^2+^-calmodulin-dependent protein kinase II (CaMKII) has been reported to be altered in atrial fibrillation and heart failure[10, 11, 21–26]. Band density was measured using ImageJ (http://imagej.nih.gov/ij/) and normalized to GAPDH.

### Ca^2+^ imaging

Atrial myocytes were re-suspended in a Tyrode’s solution containing 0.75 mM Ca^2+^ and loaded with 10 μM fluo 3-AM for 6 min at 37°C. Fluo-3-AM-loaded cells were resuspended in control Tyrode’s solution containing (in mM) 134 NaCl, 4 KCl, 1.2 MgCl_2_, 1.0 CaCl_2_, 11 D-glucose, 10 4-(2-hydroxyethyl)-1-piperazineethanesulfonic acid (HEPES; pH 7.4) and, after 30 minutes de-esterification, transferred to a bath mounted on the stage of the LSM (Zeiss, Germany). The cells were superfused with control solution and stimulated at 0.2–1 Hz by 4-ms bi-polar supra-threshold rectangular voltage pulses via a pair platinum electrodes. Experiments were performed at room temperature. Fluo-3 was excited at 488 nm and emitted fluorescence collected at wavelengths >505 nm. SR function was examined by rapid application of the RyR agonist, caffeine (10 mM). Image analysis was performed off-line using Zeiss LSM 5 Image Examiner Software V2.81 (Zeiss, Germany) and custom routines in IDL (Boulder, CO). Linescan images of electrically-stimulated ‘twitch’ transients are presented as the average of >15 consecutive transients. Fluorescence was normalized to the baseline signal (F/F_0_). Where indicated, the intracellular Ca^2+^ concentration ([Ca^2+^]_i_) was calculated using the equation:

$${\left[{Ca}^{2+}\right]}_{i}={(K}_{d}.(\frac{F}{F_{0}}))/(\frac{K_{d}}{{\left[{Ca}^{2+}\right]}_{rest}}-\frac{F}{F_{0}}+1),$$ Eq 1

assuming a dissociation constant of fluo-3 for Ca^2+^ (*K*_d_) of 400 nM and [Ca^2+^]_rest_ = 100 nM[27], where *F* represents fluorescence intensity and *F*_0_ represents the baseline *F*. This value for *K*_d_ was chosen to allow direct comparison of the data with a previous study of Ca^2+^ transport in rat atrial cells[28]. Ca^2+^ extrusion from the cytosol during the recovery of twitch and caffeine-induced transients were quantified by fitting the decaying phase of the transient with the equation:

$${{[Ca}^{2+}]}_{i}(t)={A.e}^{-k.t}+c,$$ Eq 2

where *t* represents time, *k* the rate constant, *A* a constant representing the notional peak of the transient at *t* = 0 and *c* the resting level.

### Modeling of diffusion

**Fig 1:**
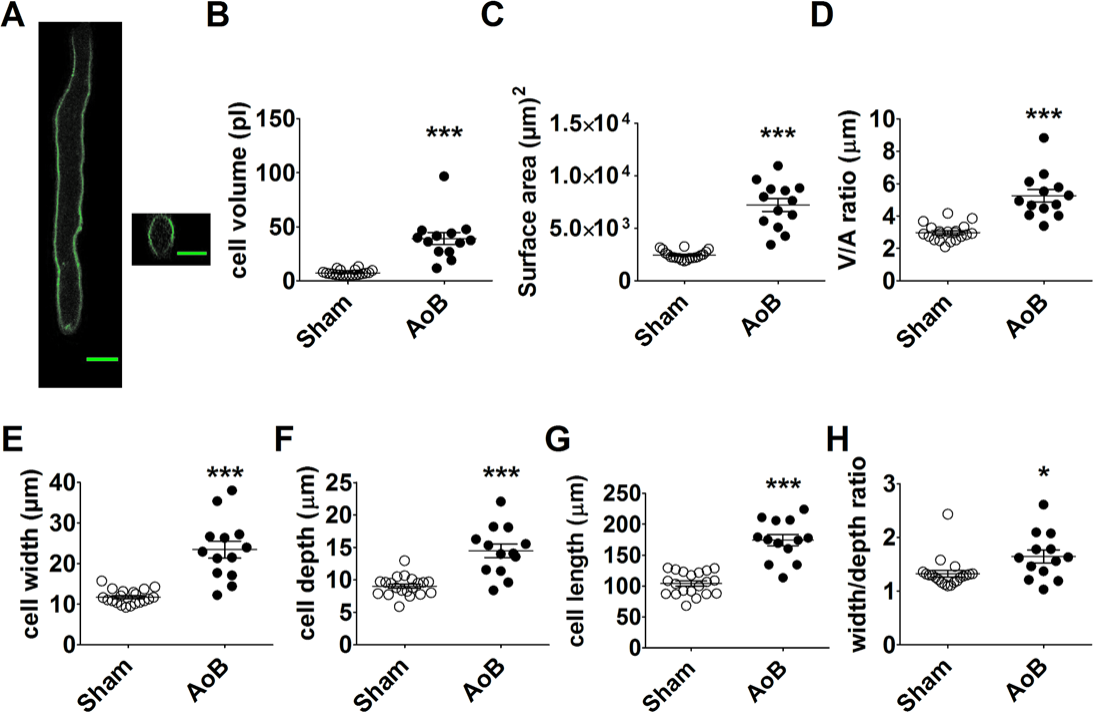
Left atrial myocyte hypertrophy. (A) left hand panel; total projected density of a series of x-y laser scanning images at various depths within an AoB left atrial myocyte stained with di-8-ANEPPS. Right hand panel; y-z section through the cell. Scale bar represents 20 μm. B–G respectively, cell volume (B), cell surface area (C), volume:surface area ratio (D), cell width (E), cell depth (F) and cell width:depth ratio of left atrial myocytes from Sham (open circles, *n*/*N* = 20/3) and AoB (filled circles, *n*/*N* = 13/3). Horizontal lines represent mean ± SEM. ###, P<0.0001, Student’s unpaired *t*-test.

To examine the gradients of Ca^2+^ that would result from diffusive processes in cells a computer model was used to solve the general reaction-diffusion equation:

$$\frac{dX}{dt}=-D_{x}.\nabla^{2}X-\sum_{B}J_{X}+J_{S},$$ Eq 3

where *D*_*X*_ is the diffusion coefficient for diffusible species *X*, *J*_*X*_ the reactive flux for all buffers *B* and *J*_*S*_ the source (and sinks) of Ca^2+^. Consideration of the elliptical cross-section of a cylindrical cell (see Fig 1A) suggested that the Laplacian should be solved in a two dimensional orthogonal elliptic coordinate system where symmetry allowed only one quadrant to be solved:

$$\nabla^{2}X=\frac{1}{a^{2}(\cosh^{2}\left(\mu )-{cos}^{2}(\nu )\right)}\left(\frac{\partial^{2}X}{{\partial \mu }^{2}}+\frac{\partial^{2}X}{{\partial \nu }^{2}}\right),\;v\in \left[0,\frac{\pi }{2}\right]$$ Eq 4

related to Cartesian cell x,y coordinates by:

x=acosh⁡(μ)+cos⁡(ν);y=asinh⁡(μ)+sin⁡(ν) Eq 5

The resulting discretization of the problem is shown in the corresponding figure. The base model major and minor axes were 10.3 and 7.6 μm respectively. Reactive fluxes were described by buffering equation:

$$J_{B}=k_{on}[Ca][B\}-k_{off}[CaB]$$ Eq 6

ATP and fluo-3 were treated as mobile buffers and troponin as a fixed buffer (for constants see Table 1).

**Table 1:** Model diffusion and buffering constants.

Species	Concentration (μM)	Diffusion coefficient (10^−10^ m^2^/s)	*k*_*on*_ (μMs^-1^)	*k*_*off*_ (s^-1^)
**Ca** ^**2+**^	0.1	3.0		
**ATP(Ca)**	0.2^a^	1.0	13.7 ^b^	3.0x10^4^
**Mg** ^**2+**^	1000	0^a^	100	31
**ATP(Mg)**	495^a^	1.0	3.3x10^-4^	3
**Fluo-3**	100	0.7	48.8	43.9
**Troponin C**	100	0	200	200

Table gives constants for the model which are based on previous cardiac simulation studies[29, 30] with minor adjustments in buffering parameters improve the fit to experimental data.

^a^ The total ATP concentration was 5 mM, which was distributed among free, Ca^2+^ bound and Mg^2+^ bound forms according to equilibrium conditions.

^b^ Diffusion of Mg^2+^ was ignored as tests showed that negligible gradients in Mg^2+^ developed.

Ca uptake by the SR Ca^2+^ pump could be distributed across the cell volume:

JSERCA=Kmax(Vmax1+(Km[Ca2+])2)−Jleak Eq 7

with *K*_*m*_ based on vesicle uptake experiments (1 μM[31]). An analogous equation was used to describe sarcolemmal Ca^2+^ extrusion with *V*_*max*_ being set to fit the observed time course of decline of the fluo-3 signal after a caffeine pulse. In the absence of an active SR pump the rate of Ca extrusion had to be increased by a factor of 170. *J*_*leak*_ is a constant to set net transport to zero for a resting [Ca^2+^] of 100 nM. Interpretation of local Ca^2+^ signals is further complicated by blurring due to the microscope point spread function, and this was simulated by convolving the model fluo-3 signal by 2D Gaussian function with full width at half maximum of 0.3 μm in x,y and 1.0 μm in z, as estimated from images of fluorescent beads and in cells[29]. Calcium signals at the edge of the cells were produced by release basis function whose parameters were varied to fit experimental time courses:

$$J_{s,t}=R_{max}(exp(-k_{off}t)(1-\exp\left({-k}_{on}t\right))$$ Eq 8

By solving the model reaction-diffusion equations and fitting results to experimental data, the requirement to deconvolve the fluorescent signals before trying to solve the inverse problem posed by the system of equations was overcome [29]. It should be noted that inverse solutions for the present problem would be (essentially) impossible due to experimental noise. Using this model we examined the spatio-temporal properties of Ca^2+^ transport across the cell to see whether Ca^2+^ signals in the center of the cell could be explained by purely passive diffusion or whether they required regenerative Ca^2+^ release.

### Statistics

Data are expressed as mean ± standard error of the mean (SEM). Data sets were subject to D’Agostino and Pearson omnibus normality test. Statistical analyses applied are indicated in the figure legends. Sample sizes are reported as *n*/*N*, where *n* = number of cells and *N* = the number of hearts. P<0.05 was considered statistically significant.

## Results

Staining of the sarcolemma of isolated left atrial (LA) myocytes from both Sham and AoB rats with the lipophilic dye, di-8-ANEPPS, was located exclusively to the cell edge, consistent with a sparsity of t-tubules in these cells[32]. From confocal volume images the cell volume (Fig 1B), surface area (Fig 1C), volume/area ratio (Fig 1D), width (Fig 1E), depth (Fig 1F) and width/depth ratio (Fig 1G) were calculated. These data show that LA myocytes from AoB rats developed marked hypertrophy in comparison with cells from Sham controls.

**Fig 2:**
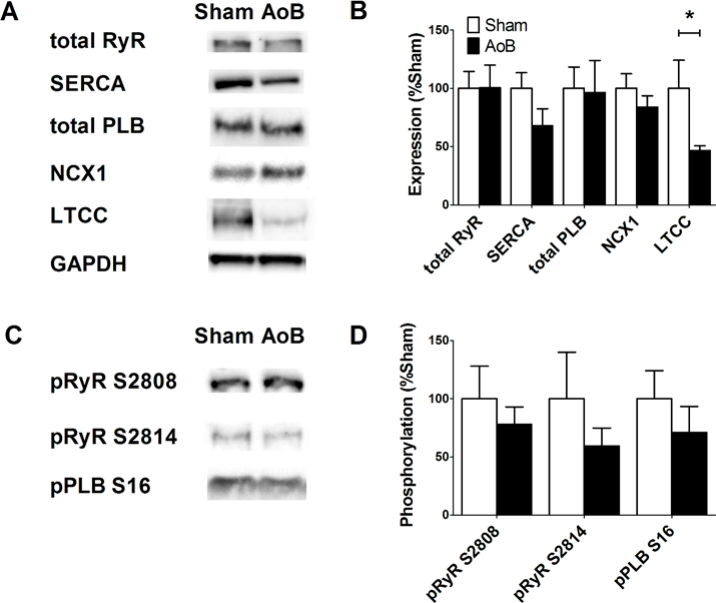
Left atrial expression of Ca^2+^ handling proteins. (A) Original Western blots of Ca^2+^ handling proteins from Sham and AoB LA. Note the GAPDH blots for normalization. (B) Mean band intensity expressed relative to Sham as 100%. Data represent mean ± SEM from 6 Sham and 6 AoB hearts. *, P<0.05, Student’s unpaired *t*-test. (C) Original Western blots of phosphorylated RyR and PLB from Sham and AoB. Data correspond to the samples shown in (A). (D) Mean band intensity expressed relative to Sham as 100%. Data represent mean ± SEM from 6 Sham and 6 AoB hearts.

The expression and phosphorylation of major proteins involved in Ca^2+^ transport was investigated by Western blotting of LA tissue homogenates from Sham and AoB rats (Fig 2). There were no significant differences between Sham and AoB in the expression of RyR2, SERCA, PLB or NCX1 (Fig 2B). On the other hand, expression of the α_1c_ subunit of LTCC was significantly reduced in AoB left atria as compared with Sham. There was no difference in the phosphorylation of RyR2 at the PKA/CaMKII sites, serine 2808 and serine 2814 or in the phosphorylation of PLB at serine 16.

**Fig 3:**
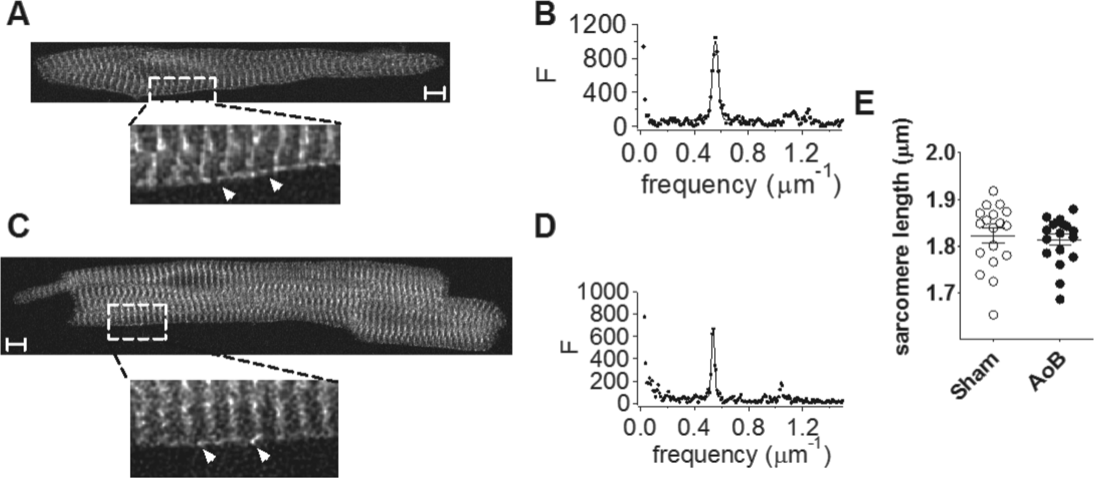
Ryanodine receptor location. (A) representative image from a Sham left atrial myocyte. Lower image, higher magnification view of area indicated by dotted box. Arrows indicate intra-sarcomeric RyR at the cell periphery. (B) power spectrum of longitudinal scan along the cell. (C) representative image from an AoB left atrial myocyte. Lower image, higher magnification view of area indicated by dotted box. (D) power spectrum of longitudinal scan along the cell. Scale bars represent 5 μm. (E) sarcomere length. Data from 18/3 Sham cells/hearts and 18/3 AoB cells/hearts.

Ryanodine receptor (RyR) labeling showed a clear striated pattern (Fig 3), as previously reported[17, 18, 33, 34]. However, there was no significant difference between Sham and AoB myocytes in the periodicity of staining of RyR2 at the sarcomeres (Sham: 0.550±0.005 μm^-1^, *n* = 18; AoB: 0.552±0.004 μm^-1^, *n* = 18; P = 0.71) or in the regularity of the staining associated with the striations (Sham: 6.0±0.8%; AoB: 4.7±0.6%; P = 0.19) as quantified by, respectively, the position and height of the peak from the Fourier transform. Western blot analysis confirmed no difference in RyR protein expression between Sham and AoB atria (Fig 2). The banded structure of the RyR labeling was consistent with the expression of RyR in corbular sarcoplasmic reticulum (SR) at the z-lines[17, 18, 33, 34]. RyR staining could be observed between the striations at the cell periphery in both Sham and AoB cells (white arrows in Fig 3A & 3B), consistent with the existence of RyR in junctional SR in close juxtaposition to the sarcolemma[17, 18, 33, 34]. Nevertheless, it is clear that the majority of RyR were not coupled to the sarcolemma. In summary, these data demonstrate that although left atrial myocytes from AoB rats had undergone significant afterload-induced hypertrophy, no remodeling of RyR expression or localization was evident.

**Fig 4:**
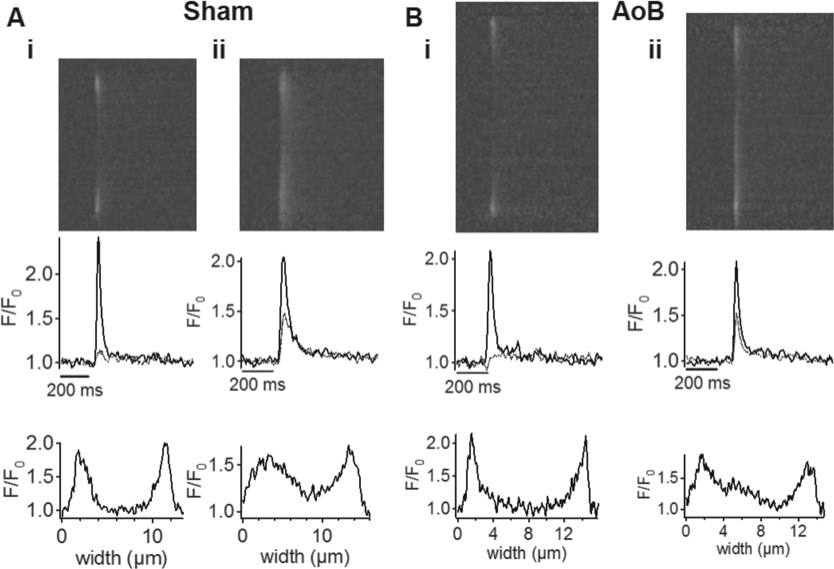
Left atrial myocyte Ca^2+^ transients in pressure-overload. (A) Upper panels: representative averaged line-scan images taken transversely across left atrial myocytes from Sham-operated rats. Middle panels: corresponding changes in [Ca^2+^]_i_ on the same time scale as the line-scan images. Black trace represents the signal at the edge of the cell, gray trace represents the signal in the center of the cell. Lower panels: transverse profile across the cell at the peak of the Ca^2+^ transient. (B) Upper panels: representative averaged line-scan images taken transversely across left atrial myocytes from AoB rats. Middle panels: corresponding changes in [Ca^2+^]_i_ on the same time scale as the line-scan images. Black trace represents the signal at the edge of the cell, gray trace represents the signal in the center of the cell. Lower panels: transverse profile across the cell at the peak of the Ca^2+^ transient. Cells were stimulated at 1 Hz.

Electrical stimulation of fluo-3-loaded cells elicited Ca^2+^-transients that were initiated at the periphery of the cells (Fig 4). Ca^2+^-transients at the edge of the cell were robust and rapidly reached a peak which was not different between the groups (F/F_0_: Sham = 2.34±0.21, *n* = 12; AoB = 2.27±0.20, *n* = 8; P = 0.99). In contrast to the cell edge, transients at the center of the cells had a much smaller amplitude (F/F_0_: Sham = 1.22±0.04, P<0.0001; AoB = 1.28±0.09, P<0.0001) and were often barely detectable (e.g. Fig 4Ai & 4Bi). The delay to the center in these cells was Sham, 39.7±6.1 ms (*n* = 10), and AoB, 50.4±21.4 ms, (*n* = 5; P = 0.54). Thus, the cells generally showed a ‘U’-shaped profile of Ca^2+^ changes across the width of the cell[35]. However, in a minority of cells (Sham 2/12; AoB 3/8; P = 0.35), significant transients could be observed at the cell center (e.g. Fig 4Aii & 4Bii). This variable pattern of release has previously been noted[36]. In summary, there were no significant differences between Sham and AoB myocytes in the amplitude of Ca^2+^ transients at the cell edge or at the cell center, nor was there any significant difference in the delay of the peak at the center of the cell relative to the cell edge.

**Fig 5:**
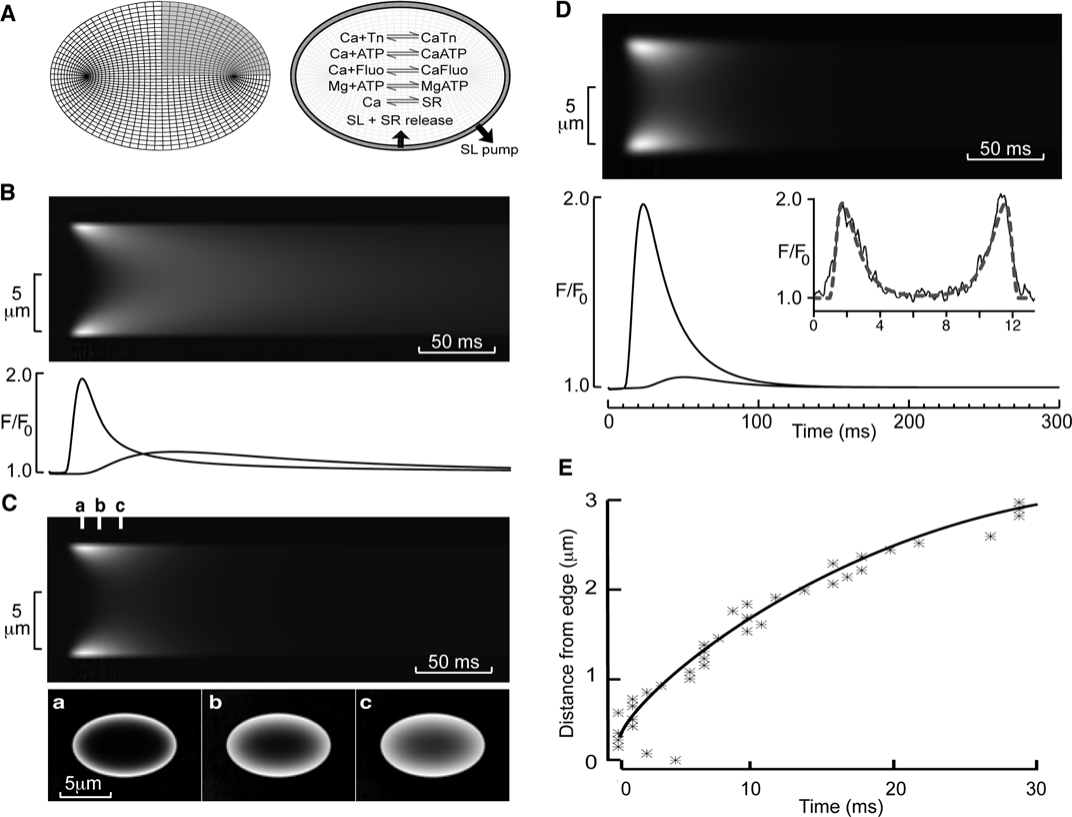
Modeling of diffusion in rat left atrial myocytes. (A) Model discretization with an elliptical grid and the Ca^2+^ reactions incorporated in the model representing Ca^2+^ buffering and fluxes. (B) Upper panel shows a simulated line scan image assuming release of Ca^2+^ only at the periphery of a cell and no *internal* Ca uptake. Lower panel shows relative fluorescence changes at the cell edge and cell center. Note the slow rate of decline of Ca^2+^ in the cell center. (C) Upper panel shows a simulated line scan image with SR Ca^2+^ uptake included. Lower panel shows the time course of fluorescence changes at the time points indicated by a, b and c in the upper panel. (D) Upper panel shows calculated line scan image including microscope blurring. Lower panel shows fluorescence changes at the edge and center of the cell model. The inset shows the radial profile at the peak of the transient for the model (dotted line) and exemplar experimental data (solid line). Note the close agreement between these data. (E) The time-dependence of spread of the peak fluorescence for the model (solid line) and exemplar experimental data (stars).

The small amplitude of the Ca^2+^ transient at the cell center clearly suggests that in the majority of cells there was little, if any, regenerative propagation of Ca^2+^ release to the center. On the other hand, the short delay between the transient at the edge and center of the cell might be consistent with the propagation of a Ca^2+^ wave involving calcium-induced calcium release[37, 38]. These divergent possibilities led us to calculate the spatio-temporal time course of simple diffusion across an elliptical cell driven by a peripheral Ca^2+^ transient (see Methods and Fig 5A). A transient increase in [Ca^2+^]_i_ to ~1 μM at the cell edge resulted in centripetal diffusion of Ca^2+^ and a significant Ca^2+^ transient occurred at the cell center (Fig 5B). However, the delay to the peak at the cell center relative to the peak of release at the cell edge (~100 ms) was considerably greater than that observed experimentally (*cf*. Fig 4). In addition, the amplitude of the transient at the cell center was greater than observed in the majority of cells. This difference between the model and experimental data could be explained by SR Ca^2+^ uptake across the cell, which, when added to the model equations, limited the effective spread of the transient to the cell center (Fig 5Ci) and reduced the time to peak and increased the rate of decline of the central Ca^2+^ transient. Adding simulated microscope blurring (measured from fluorescent beads[29]) increased the apparent speed of propagation from the cell edge to center and resulted in model data being a reasonable facsimile of experimental data (compare Fig 5D and 5E with Fig 4). Thus, the data from most cells are consistent with the idea that Ca^2+^ release occurs predominately in cell periphery via junctional RyRs with little, if any, regenerative Ca^2+^ release from the uncoupled RyRs residing in corbular SR within the cell interior.

**Fig 6:**
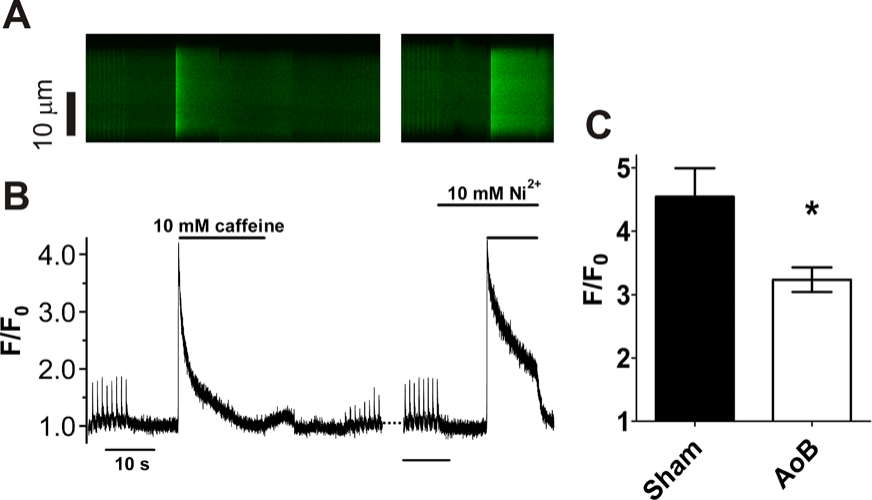
Caffeine-induced Ca^2+^ release. (A) representative line scan image of Ca^2+^ release triggered by caffeine (10 mM). Example is from a Sham myocyte. Time scale as shown in B. (B) time course of changes in spatially averaged [Ca^2+^]_i_ of the cell shown in A. Horizontal and vertical lines axes indicate [Ca^2+^]_i_ and time scale, respectively. (C) Mean (±SEM) peak F/F_0_ in the presence of caffeine in Sham (*n*/*N* = 10/5) and AoB (*n*/*N* = 13/6) cells. *, P<0.05, unpaired t-test.

SR function was examined further using the RyR agonist, caffeine (Fig 6). Caffeine (10 mM) was applied 10 s after 8 conditioning pulses at 1 Hz. The application of caffeine produced a large homogeneous release of Ca^2+^ (Fig 6A), showing that the interior SR stored releasable Ca^2+^. After the peak of release, F/F_0_ declined in the continued presence of caffeine, although more slowly than for twitch transients following electrical stimulation (Fig 6B). Following washout of caffeine, the amplitude of the Ca^2+^ transient was reduced, consistent with the loss of releasable Ca^2+^ from the junctional SR and with subsequent pulses Ca^2+^ release progressively increased in amplitude until a steady state was reached, typically after ~20 pulses. The amplitude of the caffeine-induced transient, an index of the Ca^2+^ content of the SR, was significantly smaller in AoB than in Sham myocytes (Fig 6C). This is consistent with a reduced expression of LTCC (and possibly SERCA) (Fig 2B) and a consequent decrease in Ca^2+^ influx and Ca^2+^ store uptake leading to reduced stored Ca^2+^. On the other hand, there was no notable difference between Sham and AoB myocytes in the recovery of Ca^2+^ transient amplitude following the washout of caffeine, suggesting that cytosolic Ca buffering was not increased in AoB myocytes

**Fig 7:**
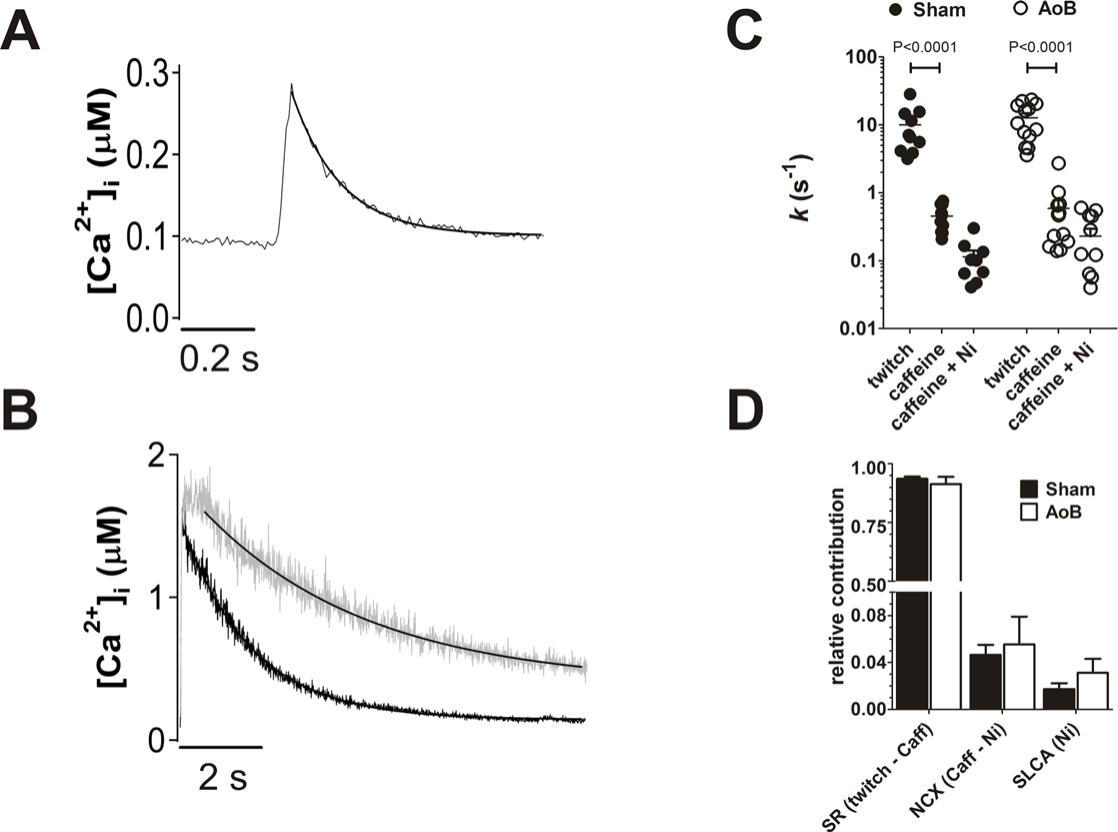
Ca^2+^ fluxes in hypertrophied atrial myocytes. (A) representative trace of twitch Ca^2+^ transient. The transient represents an average of 8 transients elicited prior to application of caffeine (see Fig 6). Solid line represents a fit to Eq 2. (B) representative traces of caffeine-induced Ca^2+^ transients in the absence (black trace) and presence (gray trace) of 10 mM Ni^2+^. Solid lines represent fits to Eq 2. (C) fitted rate constants of Ca^2+^ removal from the cytosol in Sham (filled circles, *n*/*N* = 10/5) and AoB myocytes (open circles, *n*/*N* = 13/6). Horizontal bars represent means and vertical lines show standard errors. Data correspond with those shown in Fig 6C. (D) proportions of Ca^2+^ flux via the principal transport pathways calculated from the rate constants. There were no significant differences in rate constants or proportional fluxes between Sham and AoB.

The contribution of different Ca^2+^ transporters to the extrusion of Ca^2+^ from the cytosol was examined by examining the rates of recovery of Ca^2+^ transients stimulated either electrically or by caffeine (Fig 7)[39, 40]. The rate of decline of electrically stimulated Ca^2+^ transients reflects extrusion via all pathways (i.e. the SR Ca^2+^ ATPase, NCX and sarcolemmal Ca^2+^ pump (SLCA)) (Fig 7A). In the presence of caffeine, the rate of recovery of the Ca^2+^ transient reflects sarcolemmal Ca^2+^ extrusion as the SR cannot accumulate Ca^2+^ (Fig 7B). In comparison with the electrically stimulated transient, the rate of decline of the Ca^2+^ transient in caffeine was reduced by about an order of magnitude and addition of 10 mM Ni^2+^ (to block NCX) further decreased the rate of decline of Ca^2+^ (Fig 7B) by a factor of ~3. There were no significant differences between Sham and AoB myocytes in the rate constants of Ca^2+^ extrusion during either twitch or caffeine-induced transients (Fig 7C) or in the presence of Ni^2+^ (Fig 7C), consistent with the absence of changes in expression of SERCA and NCX1 proteins (Fig 2). The relative contribution of the transport pathways to Ca^2+^ extrusion were calculated from the rate constants[41] and no significant difference between Sham and AoB myocytes were found (Fig 7D).

**Fig 8:**
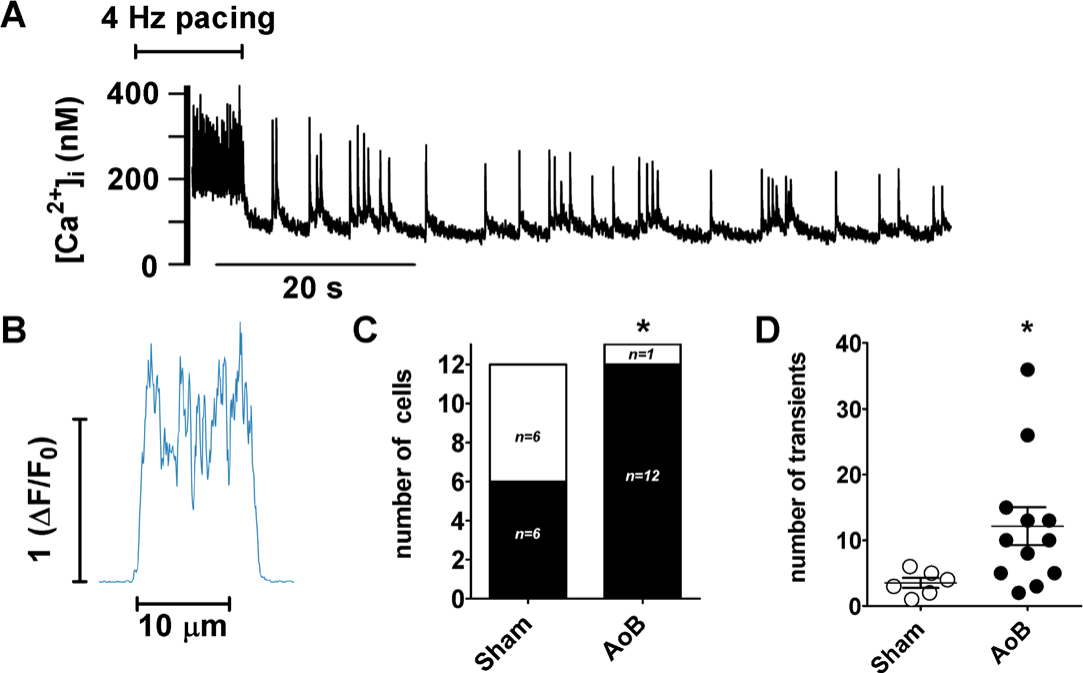
Spontaneous Ca^2+^ transients in remodeled left atrial myocytes. (A) Spatially averaged [Ca^2+^]_i_ obtained from an AoB cell during a period of pacing at 4 Hz followed by 70 s without stimulation. The resolution in these experiments was insufficient to localize the sites of release. (B) Averaged transverse profile of spontaneous Ca^2+^ transients from the cell shown in ‘A’. (C) Shaded area indicates number of Sham and AoB myocytes showing spontaneous Ca^2+^ transients after 4 Hz pacing; height of column represents total number of myocytes tested. Data from 4 Sham and 6 AoB hearts. *, P<0.05, Fischer’s exact test. (D) Number of spontaneous transients in cells showing spontaneous activity after pacing at 4 Hz. *, P<0.05, unpaired t-test.

In order to examine the impact of afterload-induced hypertrophic remodeling on susceptibility to spontaneous Ca^2+^ release, myocytes were subject to rapid pacing (Fig 8). Following rapid pacing, spontaneous Ca^2+^ transients occurred in both Sham and AoB myocytes (e.g. Fig 8A). The averaged profile of the spontaneous Ca^2+^ transients indicated that Ca^2+^ release was not confined to a particular location within the cell (Fig 8B). However, the proportion of cells showing spontaneous transients was significantly higher in AoB cells (92%) compared with Sham myocytes (50%; Fig 8C). In addition, the number of spontaneous transients observed per cell was greater in AoB cells than in Sham (Fig 8D).

## Discussion

This study shows that increased afterload leads to atrial cell hypertrophy and an increased susceptibility to spontaneous Ca^2+^ transients. This mimics the reported increased atrial arrhythmia risk in hypertensive patients (e.g.[2]). In view of the central role of Ca^2+^ metabolism in the generation of spontaneous diastolic electrical activity[8], we examined Ca^2+^ transport and RyR distribution in the aortic banded model. Despite a large change in cell dimensions, Ca^2+^ transport and RyR distribution were hardly affected. There were no changes in the expression of RyR nor in RyR phosphorylation at the PKA/CaMKII sites, S2808 and S2814 (Fig 2) [21]. On the other hand, the amplitude of the caffeine-induced Ca^2+^ transient, an index of SR Ca^2+^ content, was reduced without any detectable change in transport via SERCA or NCX. Taken together, our data are consistent with reduced LTCC expression, presumably resulting in decreased Ca^2+^ influx, in elevated afterload-induced atrial cellular remodeling.

### Spatial heterogeneity in SR Ca^2+^ release

Myocytes exhibited marked non-uniformity in the Ca^2+^ transient across the width of the cell (e.g. lower panels of Fig 4Ai and 4Bi). By modeling 2D centripetal diffusion in an elliptical cell cross-section we showed that active SR Ca^2+^ uptake across the cell is essential to produce the spatio-temporal patterns of Ca^2+^ release observed. To our knowledge, this is the first report of such 2D modeling and shows that optical blurring by the microscope must also be considered to interpret correctly the spatio-temporal movements of Ca^2+^ across the cell width[29], and due to the cell ellipticity, reduction of the problem toward circular cylindrical models or even one dimension (as previously used in other studies) is likely erroneous.

The modeling shows that without SR Ca^2+^ uptake across the cell, the time course of decline of the internal Ca^2+^ transient is ~10 times slower (Fig 5B), showing that internal SR Ca^2+^ uptake plays a key role in making sure that the Ca^2+^ transient at the center of the cell is of comparable duration to that seen at the periphery. However, since little SR release occurs from the deeper SR (despite the presence of RyRs), the Ca^2+^ that is taken up must return to the cell surface by diffusion within the SR lumen (for subsequent release and/or extrusion), a process reminiscent of the SR vectorial transport and buffer barrier described for smooth muscle cells[42]. This will have been particularly significant following rapid pacing in the present study, when it can be expected that the SR would become loaded, particularly within the deeper SR, and this may contribute to the incidence of spontaneous Ca^2+^ release events in Sham cells (Fig 8) if the deep SR becomes overloaded (see also below).

### Atrial Ca^2+^ regulation in hypertrophy

The present study is the first explicitly to examine Ca^2+^ regulation in a purely afterload-induced atrial hypertrophy. Spontaneous Ca^2+^ release has been reported to be increased in AF in association with changes in RyR function, although SR Ca^2+^ content was reported to be unchanged and Na^+^/Ca^2+^ exchange function increased[9–11, 43]. Reduced expression of the LTCC α_1c_ subunit has also been reported in human AF, associated with a decreased L-type Ca^2+^ current density [13, 15, 44]. Increased susceptibility to spontaneous Ca^2+^ release has been shown to lead to atrial arrhythmogenesis in a canine model of heart failure [23]. The increased susceptibility to spontaneous atrial Ca^2+^ release in heart failure has been associated with increased SR Ca^2+^ load despite reduced LTCC expression and Ca^2+^ current[23, 45, 46]. Our results contrast with those of a recent study of atrial cellular remodeling in a rat model of essential hypertension in which spontaneous Ca^2+^ release was *not* increased in atrial myocytes despite an increased SR Ca^2+^ load [47]. While, as found in the present study, LTCC expression was reduced, in contrast to our findings, expression of RyR2 and NCX1 was also reduced and phosphorylation of RyR2 at S2808 was increased[47]. In the present study, the amplitude of the electrically stimulated Ca^2+^ transient was not different between AoB and Sham myocytes, despite the reduced SR Ca^2+^ load and Ca^2+^ trigger via LTCC. That atrial effective refractory period has been reported to be unaffected in AoB hearts indicates that atrial action potential duration was unchanged[4]. Thus, these observations indicate that cytosolic buffering of Ca^2+^ was reduced in AoB cells, as has been recently reported in an ovine model of heart failure[46]. A reduction in cytosolic buffering would have diminished the difference between Sham and AoB cells in amplitude of the caffeine-induced transient. For example, in the absence of changes in SR Ca^2+^ load, a reduction in cytosolic buffering would have resulted in an *increase* in the amplitude of the caffeine-induced transient. Thus, the reduced amplitude of the caffeine-induced Ca^2+^ transient in AoB compared with Sham cells in the present study provides robust evidence of a reduction in atrial SR Ca^2+^ load through elevated afterload-induced remodeling.

The absence of differences in the present study between Sham and AoB in SERCA and NCX expression was reflected in the lack of difference in the rate constants of recovery of twitch and caffeine-induced Ca^2+^ transients, demonstrating that the activity of the major Ca^2+^ extrusion systems, the SR Ca^2+^ ATPase and the Na^+^/Ca^2+^ exchanger, were not affected by the afterload-induced atrial hypertrophy. Moreover, the values of *k*_twitch_ and *k*_Caff_ were similar to those reported by Trafford and colleagues in normal atrial myocytes[28].

Given the key role played by SR luminal Ca^2+^ in regulating spontaneous release [48], it is notable that the increased susceptibility to spontaneous Ca^2+^ release in AoB compared with Sham atrial cells in the present study occurred despite reduced SR Ca^2+^ load. Presumably, the increased incidence of spontaneous release events in AoB cells reflects the increased volume of the cell: As there was no change in RyR distribution in the hypertrophied cells, the number of RyRs and potential release sites must have been increased across the cell (Fig 3), with the consequence that, even without a change in the RyR gating probability, the incidence of spontaneous release events following rapid pacing would be increased. Consistent with this proposal, the mean number of spontaneous transients per cell, including those cells that did not show spontaneous Ca^2+^ transients, was increased ~6.4-fold in AoB compared to Sham cells, a figure that corresponds well with the ~5.3-fold increased cell volume of AoB relative to Sham cells.

In summary, we have shown in an aortic banded model in rats that elevated afterload causes atrial cellular hypertrophy in which cellular Ca^2+^ transport and RyR2 distribution were unaffected but there was an increased susceptibility to spontaneous Ca^2+^ release, presumably reflecting maladaptive changes in cell geometry. We suggest that the increased susceptibility to spontaneous Ca^2+^ release reported here may underly the increased incidence of atrial tachyarrhythmias in conditions of elevated afterload.
